# Prophylactic infusion of donor derived CMV specific T cells for the prevention of CMV reactivation following allogeneic HSCT

**DOI:** 10.1038/s41598-025-27354-6

**Published:** 2025-12-04

**Authors:** Maryam Barkhordar, Maryam Samareh Salavatipour, Shirin Tavakoli, Tanaz Bahri, Marjan Yaghmaie, Mohammad Vaezi, Leyla Sharifi Aliabadi, Ghasem Janbabai, Mina Naderi, Mohammad Ahmadvand

**Affiliations:** 1 Hematologic Malignancy Research Center, Research Institute for Oncology, Hematology and Cell Therapy, Tehran University of Medical Sciences, Tehran, Iran; 2 Department of Applied Cell Sciences, School of Advanced Technologies in Medicine, Tehran University of Medical Sciences, Tehran, Iran; 3 Cell Therapy and Hematopoietic Stem Cell Transplantation Research Center, Research Institute for Oncology, Hematology and Cell Therapy, Tehran University of Medical Sciences, Tehran, Iran; 4 Research Institute for Oncology, Hematology and Cell Therapy, Tehran University of Medical Sciences, Tehran, Iran; 5 Department of Hematology-Oncology, Imam Hossein Hospital, School of Medicine, Shahid Beheshti University of Medical Sciences, Tehran, Iran

**Keywords:** Cytomegalovirus, Allogeneic hematopoietic stem cell transplantation, Adoptive cell therapy, GVHD, Virus-specific T cells, Cancer, Immunology, Medical research

## Abstract

Cytomegalovirus (CMV) reactivation remains a common and serious complication following allogeneic hematopoietic stem cell transplantation (allo-HSCT), often leading to significant morbidity and mortality in immunocompromised recipients. Adoptive transfer of CMV-specific cytotoxic T lymphocytes (CMV-CTLs) has emerged as a promising immunotherapeutic approach to restore antiviral immunity and prevent CMV-related complications. This interim analysis of a randomized phase I–II clinical trial evaluates the safety and preliminary efficacy of prophylactic infusion of donor-derived CMV-CTLs administered early after allo-HSCT. Twenty adult allo-HSCT recipients were randomized 1:1 to receive either standard care (control group) or a single intravenous infusion of 10 million donor-derived CMV-CTLs per m^2^ of body surface area (intervention group) between days + 14 to + 21 post-transplant. The primary endpoint was safety, defined by infusion-related adverse events and incidence of acute graft-versus-host disease (aGvHD). Secondary endpoints included incidence and kinetics of CMV reactivation, measured by serial quantitative RT-PCR during the first 90 days post-transplant. Baseline characteristics were balanced between the two groups. No grade 3–5 adverse events were observed following CMV-CTL infusion. aGvHD grade II occurred in 30% of the intervention group versus 40% in controls. The incidence of CMV reactivation at 90 days was lower in the intervention group (52.00%) compared to controls (77.78%), although not statistically significant (P = 0.580). However, CMV viral loads were significantly lower over time in the intervention group (P = 0.028), suggesting a favorable antiviral effect of CMV-CTLs. Prophylactic infusion of donor-derived CMV-CTLs early after allo-HSCT appears safe and may reduce CMV reactivation and viral burden. These findings support the potential of CMV-CTLs as a novel immunotherapeutic strategy for CMV management in high-risk transplant recipients. Further validation in larger, multicenter trials is warranted.

**Supplementary Information:**

The online version contains supplementary material available at 10.1038/s41598-025-27354-6.

## Introduction

Cytomegalovirus (CMV), a β-herpesvirus with global seroprevalence rates ranging from 44 to 100%, establishes lifelong latency and poses a major threat to immunocompromised individuals^1^. While asymptomatic in immunocompetent hosts, in allogeneic hematopoietic stem cell transplantation (allo-HSCT) recipients, profound immunosuppression disrupts T cell–mediated antiviral defense, leading to CMV reactivation in up to 70% of seropositive patients within the first 100 days. Such reactivation is a leading cause of morbidity and contributes to a 20–30% increase in transplant-related mortality, primarily through complications such as pneumonitis, colitis, and bone marrow suppression^2–5^.

Conventional antiviral agents such as ganciclovir and valganciclovir are associated with hematologic toxicities, including neutropenia and anemia, which may complicate post-transplant management. In contrast, letermovir has emerged as a highly effective and well-tolerated prophylactic agent against CMV reactivation in allogeneic transplant recipients, as supported by recent consensus and clinical trial data. Nonetheless, despite its favorable safety profile, the high cost and limited availability of letermovir remain significant challenges in many healthcare settings, particularly in resource-limited countries^6^. These shortcomings underscore the need for novel strategies. Adoptive transfer of donor-derived CMV-specific cytotoxic T lymphocytes (CMV-CTLs) has thus emerged as a promising approach to reconstitute antiviral immunity while minimizing off-target effects^7–9^. CMV-CTLs are generated by ex vivo expansion of donor T cells primed with immunodominant CMV antigens (e.g., pp65, IE-1), enabling rapid reconstitution of antiviral immunity post-infusion^10,11^.

Clinical trials demonstrate that prophylactic or therapeutic CMV-CTLs safely reduce reactivation rates, even in antiviral-refractory cases, while minimizing graft-versus-host disease (GvHD) risk through antigen-specific targeting^12–14^. For example, a phase I/II trial of CMV-TCR-T cells in patients with haploidentical donor transplants showed feasible safety and considerable suppression of viral reactivation^8^. These data highlight that T-cell therapy not only treats existing CMV reactivation but may also prevent disease onset in high-risk patients. In contexts where antiviral prophylaxis (for instance, letermovir) is inaccessible or the costs may be prohibitive in countries with socioeconomic healthcare issues (such as Iran), reliance on competent cellular immunity becomes indispensable^15,16^. Thus, early infusion of CMV-specific T cells represents a clinically meaningful supplement or alternative, especially in settings with limited access to newer antivirals.

Building on this evidence, we conducted a randomized trial evaluating early prophylactic infusion of donor-derived CMV-CTLs administered shortly after allo-HSCT. Our study aims to address a critical unmet need in transplant recipients, particularly in healthcare settings where access to novel antivirals such as letermovir is limited.

## Methods

### Study design

We conducted a randomized phase I/II trial at a single center to assess the safety and preliminary efficacy of donor-derived CMV-specific cytotoxic T lymphocytes (CMV-CTLs) in preventing CMV reactivation after allogeneic hematopoietic stem cell transplantation (allo-HSCT). The trial followed CONSORT guidelines, received approval from the Institutional Ethics Committee of Hematology-Oncology and Stem Cell Transplantation Research Center, Tehran University of Medical Sciences (Approval ID: IR.TUMS.HORCSCT.REC.1401.015), and was registered with the Iranian Registry of Clinical Trials (IRCT20140818018842N30) on 18/02/2023. All procedures adhered to the Declaration of Helsinki. This report summarizes interim findings for Part 1 of the study, with final results to be separately reported.

### Patient recruitment and randomization

From February to July 2023, we screened adult leukemia patients (≥ 18 years) scheduled for allo-HSCT at Tehran University of Medical Sciences. To qualify, patients needed to be CMV-seropositive recipients with CMV-seropositive donors, a high-risk group for reactivation. We excluded those receiving grafts from unrelated donors or participating in other trials. Using a balanced block design, we randomly assigned patients 1:1 to either standard care (control) or a single CMV-CTL infusion (10 × 10^6^ cells/m^2^) administered between days + 14 and + 21 post-transplant. During the patient enrollment period in our institution, letermovir was not available; therefore, CMV management in both arms followed a pre-emptive strategy-based standard of care preemptive therapy including ganciclovir/valganciclovir.

### CMV monitoring & laboratory testing

Plasma CMV DNAemia was monitored by quantitative real-time PCR (qPCR; COBAS® AmpliPrep/COBAS® TaqMan, Roche Diagnostics) at weekly intervals. Preemptive antiviral therapy was initiated at > 500 copies/mL per institutional protocol. All PCR assays were performed in a single reference laboratory to ensure inter-assay consistency. Upon a positive result for viral DNA levels, preemptive ganciclovir/valganciclovir therapy is initiated to manage CMV reactivation.

### Procedures

#### Conditioning and GvHD prophylaxis

All patients received myeloablative conditioning with intravenous busulfan (3.2 mg/kg/day from days − 6 to − 3) and cyclophosphamide (60 mg/kg/day on days − 3 and − 2). To prevent graft-versus-host disease (GvHD), they received cyclosporine (1.5 mg/kg/day IV starting day + 5) and methotrexate (10 mg/m^2^ on day + 1, followed by 6 mg/m^2^ on days + 3, + 6, and + 11). For haploidentical transplants, we added antithymocyte globulin (ATG; 2.5 mg/kg/day on days − 3 to 1) and post-transplant cyclophosphamide (PTCy; 40 mg/kg/day on days + 3 and + 4).

#### CMV-specific T cell production

We generated CMV-CTLs by stimulating donor peripheral blood mononuclear cells (PBMCs) with GMP-grade peptide mixes (JPT Technologies) targeting CMV antigens pp65 and IE-1. These 15-mer peptides overlapped by 11 amino acids to ensure broad immune coverage. Cells were cultured in RPMI-1640 media supplemented with IL-7 (10 ng/mL) and IL-4 (400 U/mL), with media refreshed on day 5. After 12 days, we harvested viable cells, confirmed their phenotype (≥ 98% CD3^+^), and cryopreserved them for infusion. Phenotypic characterization of expanded CMV-specific T cells was performed by flow cytometry using fluorochrome-conjugated anti-CD3 and anti-CD45 antibodies. Cells were gated on lymphocyte populations by forward/side scatter, and CD3 + expression was confirmed with ≥ 90% purity before release. Viability was assessed using 7-AAD exclusion. For cryopreservation, cells were resuspended at 1–2 × 10^7^ cells/mL in CryoStor® CS10 (STEMCELL Technologies) containing 10% DMSO and frozen in controlled-rate freezing containers (Mr. Frosty, Nalgene) at –80°C overnight before transfer to liquid nitrogen vapor phase storage. Thawing was performed rapidly at 37°C, followed by washing in RPMI + 10% FBS to remove DMSO.

### CMV-specific T-cell lines generation

#### Pepmixes

For PBMC stimulation, we used pepmixes (15mers overlapping by 11aa) spanning CMV-pp65, IE-1. These custom Good Manufacturing Practices (GMP) grade pepmixes were synthesized by JPT Technology. Each pepmix was vialed at a concentration of 25 µg/peptide/vial, and for reconstitution, each vial was resuspended in 125 µl of Dimethyl sulfoxide (DMSO). To generate the pepmix master mix, equivalent volumes of each pepmix were mixed together, aliquoted, and stored at -80 °C.

#### VST generation

To generate VSTs, 15 × 10^6^ fresh PBMCs were pelleted in a 15 ml tube and pulsed for 30–60 min at 37 °C with the pepmix mastermix at a concentration of 100 ng/peptide/15 × 10^6^ PBMCs. After incubation, the cells were resuspended in 30 ml of VST media [Advanced RPMI 1640 (Life Technologies) supplemented with 45% Click’s medium (Irvine Scientific), 2 mM GlutaMAX (Life Technologies), and 10% Fetal Bovine Serum (Hyclone)] supplemented with 10 ng/ml IL-7 and 400 U/ml IL-4 (both from Miltenyi Biotech) and plated out in a 24-well plate (2 × 10^6^/well). Media and cytokines were replenished on day 5, and cultures were split when they reached a density > 3 × 10^6^ cells/24-well. On day 11–12, CTLs were harvested, counted, and used for phenotypic and functional studies. On days 11–12, VSTs were harvested and counted to assess viability using trypan blue exclusion. Appropriate samples were sent for quality control testing as well as for phenotypic and functional studies, and the remainder was cryopreserved for clinical use.

#### Administration and monitoring of CTL infusion

Premedication for CTL infusion, including acetaminophen up to 650 mg (or 10 to 15 mg/kg recipient weight) and diphenhydramine up to 50 mg (or up to 0.5 to 1 mg/kg recipient weight), is given orally or IV before CTL infusion and repeated 4 h later. Steroids were avoided as they interfered with the efficacy of the infused CMV-specific CTLs, and it was given if the patient developed a severe or life-threatening reaction to the infused CMV-specific CTLs. The CTL product was given as a single dose of systemic intravenous infusion of 10 million cells/m2 CMV-specific T-cells after day 14 post-transplant.

### Outcomes

The primary endpoint was the safety of the CMV-CTL infusion, including severe infusion-related adverse events (graded by CTCAE v5.0) and acute GvHD incidence within 100 days. Since the secondary endpoint was assessing the efficacy of preventing CMV reactivation efficacy of the CMV-CTL infusion in preventing CMV reactivation was evaluated by measuring CMV reactivation using quantitative reverse transcription-polymerase chain reaction (RT-PCR) at weekly intervals for three months following allo-SCT. The CMV RT-PCR levels were assessed and compared between the intervention and control groups.

### Statistical analysis

We initially planned to enroll 40 patients to detect a 30% reduction in CMV reactivation with 80% power (α = 0.05). For this interim analysis, descriptive statistics were employed to summarize the data, with quantitative variables reported as mean with standard deviation (SD) and median with interquartile range (IQR). In contrast, qualitative variables were expressed as frequency with percentage. The characteristics of the patients in the control and intervention groups were analyzed to determine any potential confounding factors. The recipient and donor age, recipient and donor gender, ABO compatibility, background disease, remission status pre-HSCT, HSCT type, CD3 and CD34 cell counts, and donor relation were compared between the two groups.

Cumulative incidence analysis for CMV reactivation was calculated and its changes over time were assessed using a Mixed-effects model between two groups. A p-value < 0.05 was reported as statistically significant. All statistical analyses were conducted using STATA version 17 (StataCorp, LP, College Station, TX, USA). GraphPad Prism software, version 9.5.1 (GraphPad Software Inc., San Diego, CA, USA), was used for the graphical presentation.

## Results

### Patients’ characteristics

From February 2023 to July 2023, twenty adult allo-SCT recipients were included in the study, with 10 patients in the intervention group receiving a single dose of 10 million cells/m^2^ CMV-CTLs and 10 patients in the control group. Furthermore, a total of 12 patients required preemptive antiviral treatment (7 in the control arm, 5 in the intervention arm).

As illustrated in Tables 1 and 2, baseline characteristics of the patients in both study arms were similar, with no significant differences observed. The median follow-up period was 14.49 months for the overall study population, 14.54 months for the intervention group, and 14.26 months for the control group.

**Table 1:** Baseline characteristics of patients undergoing hematopoietic stem cell transplantation (HSCT). This table summarizes demographic data, underlying diseases, conditioning regimens, and donor types for all study participants.

Characteristics		Control group	ATC group
Number of participants		10	10
Recipient age in years, Median (Range)		33 (20–47)	35.5 (18–51)
Donor age in years, Median (Range)		39 (10–55)	34 (19–53)
Recipient gender	Female	4 (40%)	6 (60%)
Donor gender	Female	6 (60%)	7 (60%)
ABO compatibility	Matched	6 (60%)	6 (60%)
	Mismatched	4 (40%)	4 (40%)
Background disease	AML	6 (60%)	5 (50%)
	ALL	4 (40%)	5(50%)
Remission status pre HSCT	CR1	4 (60%)	6 (60%)
	CR > = 2	6 (40%)	4 (40%)
HSCT type	Haploidentical	7 (70.0%)	7 (70.0%)
	Full matched	3 (30.0%)	3 (30.0%)
CD3*10^6^, mean (± SD)		333.55 (± 88.02)	250.13 (± 76.50)
CD34*10^6^, mean (± SD)		8.64 (± 1.66)	6.54 (± 1.54)
Donor relation	Sibling	7 (70.0%)	10 (100%)
	Parents	1 (10%)	0 (0%)
	Offspring	2 (20)	0 (0%)
Disease risk index	Low/Intermediate	3 (30%)	3 (30.0%)
	Intermediate/High	7 (70%)	7(70%)

Data are presented as frequency (%), Median (Range), and mean (± standard deviation).

ATC, Adaptive T Cell; HSCT, Hematopoietic Stem Cell Transplantation; CR, Complete Response; ALL, Acute Lymphoblastic Leukemia; AML, Acute Myeloid Leukemia; SD, standard deviation.

**Table 2:** Individual patient characteristics and clinical outcomes by study arm.

Id	Arm	R. Age	D. Age	R-D Sex matching	ABO Matching	Prim Dis	Remission status Pre HSCT	HSCT Type	CMV Reactivation	Max titer CMV	Duration	Acute GVHD grading	Survival status	Cause of death	Follow-up time by days
1	ATC	27	36	F-F	Matched	AML	CR1	Haplo	+	1200	35	2	Alive	-	472
2	Control	29	39	M-F	Bidirectional	AML	CR > = 2	MRD	-	0	0	2	Alive	-	476
3	ATC	25	38	M-F	Minor MM	ALL	CR > = 2	Haplo	-	0	0	0	Alive	-	460
4	Control	31	32	F-F	Matched	AML	CR1	MRD	+	1100	65	2	Alive	-	443
5	ATC	51	43	F-F	Matched	ALL	CR > = 2	Haplo	+	610	20	0	Alive	-	453
6	ATC	44	53	F-F	Minor MM	AML	CR1	Haplo	-	621	10	2	Death	Infection (pseudomonas)	151
7	ATC	34	28	F-F	Matched	ALL	CR1	MRD	-	0	0	2	Death	GvHD	453
8	ATC	44	44	M-F	Matched	ALL	CR1	Haplo	+	820	25	0	Alive	-	446
9	ATC	34	29	M-M	Matched	ALL	CR1	Haplo	-	0	0	1	Alive		404
10	Control	47	20	M-M	Matched	AML	CR1	Haplo	+	1650	30	1	Alive	-	425
11	Control	20	49	M-F	Matched	ALL	CR > = 2	Haplo	+	1200	25	1	Alive	-	367
12	ATC	18	19	M-F	Minor MM	AML	CR > = 2	Haplo	+	1750	40	0	Alive	-	439
13	ATC	45	31	F-M	Major MM	AML	CR1	MRD	-	0	0	0	Alive	-	366
14	ATC	37	32	F-M	Matched	AML	CR > = 2	MRD	+	1083	14	0	Alive	-	401
15	Control	40	55	F-M	Matched	AML	CR > = 2	Haplo	+	3000	40	0	Death	Infection (Aspergillosis)	95
16	Control	24	14	M-F	Minor MM	ALL	CR > = 2	Haplo	-	800	20	0	Alive	-	453
17	Control	35	42	F-M	Matched	AML	CR > = 2	Haplo	-	0	0	1	Alive	-	480
18	Control	35	10	M-F	Matched	ALL	CR1	Haplo	+	720	45	2	Death	Relapse	289
19	Control	28	39	M-F	Minor MM	ALL	CR > = 2	Haplo	+	1150	30	2	Death	Infection (unknown)	163
20	Control	38	49	F-M	Major MM	AML	CR1	MRD	+	940	45	0	Alive	-	474

ATC, Adaptive T Cell; R, Recipient; D, Donor; Prim Dis, Primary Disease; HSCT, Hematopoietic Stem Cell Transplantation, MM, Miss Match; F, Female; M, Male; ALL, Acute Lymphoblastic Leukemia; AML, Acute Myeloid Leukemia Match; CR, Complete Response; MRD, Match Related Donor; Haplo, Haploidentical.

### CMV-specific T cell expansion

**Fig. 1:**
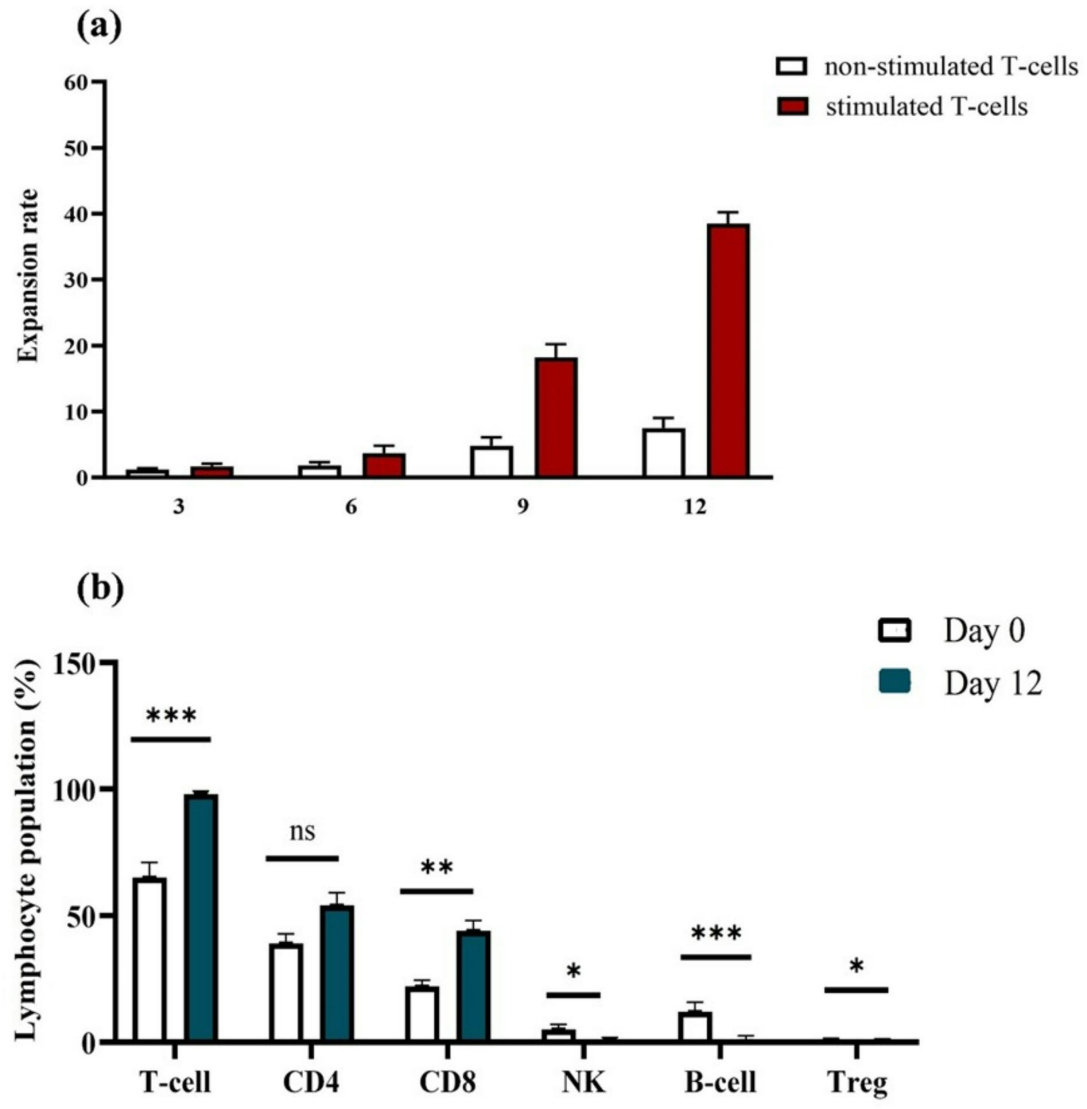
The expansion of T cells specific to pp65 and IE1. (**a**) The expansion rate of VSTs across various time points during a 12-day culture involving PBMC co-cultured with peptide pool-stimulated PBMC and non-stimulated PBMC. (**b**) The percentage of distinct cell subpopulations before (day 0) and after expansion (day 12).

Donor PBMCs stimulated with pp65/IE-1 peptides expanded 38.9-fold over 12 days, yielding a median of 4.5 × 10^8^ cells per product (range: 1.1–6.8 × 10^8^) (Fig. 1a). The final product comprised predominantly CD3 + T cells (98.1 ± 1.2%), with balanced CD4 + (54.3 ± 5.9%) and CD8 + (44.8 ± 5.2%) subsets (Fig. 1b). Non-T cell populations (NK, B cells, Tregs) were nearly undetectable post-expansion.

As shown in Fig. 1b, the final product was primarily composed of T cells, with a significant predominance of CD3^+^ T cells (mean, 98.1 ± 1.2%), encompassing both CD4^+^ (mean, 54.3 ± 5.9%) and CD8^+^ (mean, 44.8 ± 5.2%) populations, alongside various other cell types including NK cells, B cells, and Treg cells. Characteristics of infused CTL products are shown in Fig. 1b. In comparison to the phenotype of the original product, notable differences were observed in the CD3^+^ cell subset, which had significantly expanded (P < 0.001). At the same time, there were no alterations in the CD4^+^ cell subset (P > 0.05) but a significant rise in the CD8^+^ cell population (P < 0.01). The expansion of B cells, NK cells, and Treg cells was absent, leading to their presence dropping to nearly undetectable levels in the final product.

### CTL infusion

A total of 10 patients were eligible to receive 10 million cells/m^2^ CMV CTL after day 14 post-transplant. Participants’ characteristics are shown in Table 1. For every participant in the study, we successfully established CMV-specific T-cell lines and expanded the cellular populations to the requisite quantities as delineated in our experimental design (10 × 10^6^/m^2^). Within the control cohort, seven individuals received transplants utilizing stem cells from their HLA-identical sibling donors. In contrast, one patient (10%) was transplanted with stem cells derived from parents, and the remaining two patients (20%) received grafts that exhibited mismatched compatibility (from offspring). Conversely, all patients (100%) received transplants from matched related sibling donors in the intervention cohort.

### CMV reactivation and viral load kinetics

**Fig. 2:**
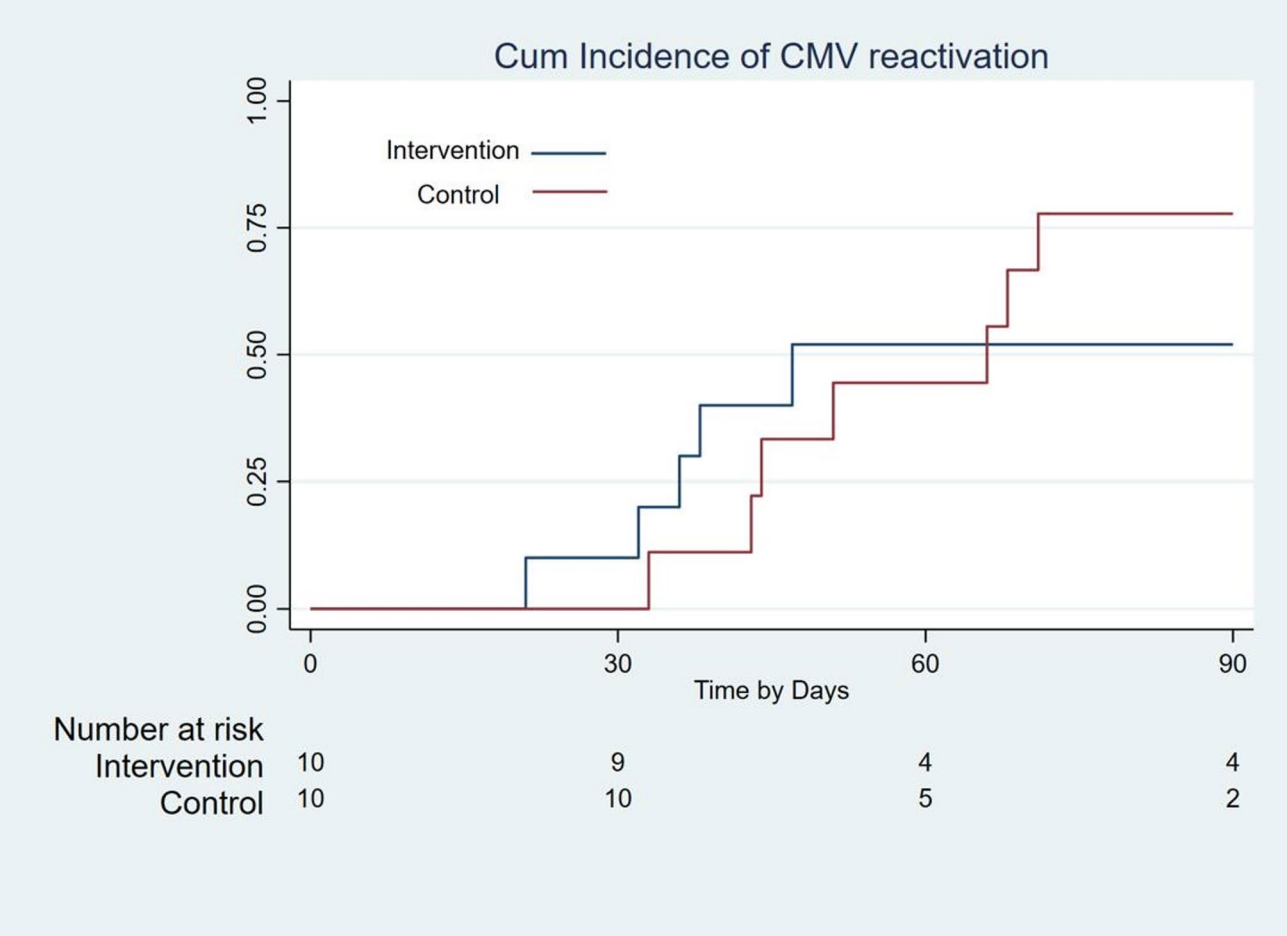
Incidence of CMV reactivation at 90 days post-HSCT in the intervention and control groups. Incidence of CMV reactivation at 90 days post-HSCT in the intervention and control groups. Two patients in the intervention group had low-level viremia prior to infusion; however, their viral loads were below the preemptive therapy threshold of 500 copies/mL.

**Fig. 3:**
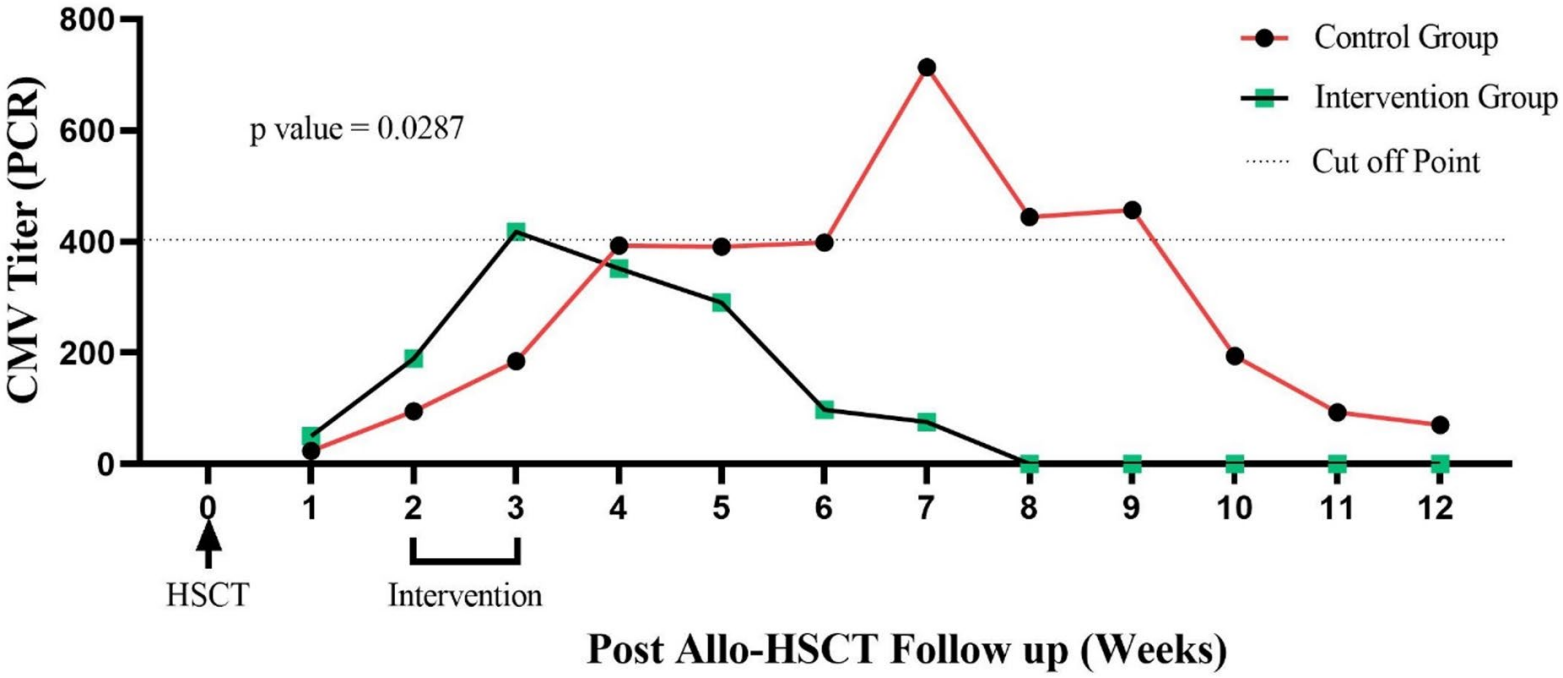
Dynamic changes in CMV viral load over time, as measured by quantitative RT-PCR. The intervention group exhibits significantly lower CMV viral loads compared to the control group (p = 0.028).

**Fig. 4:**
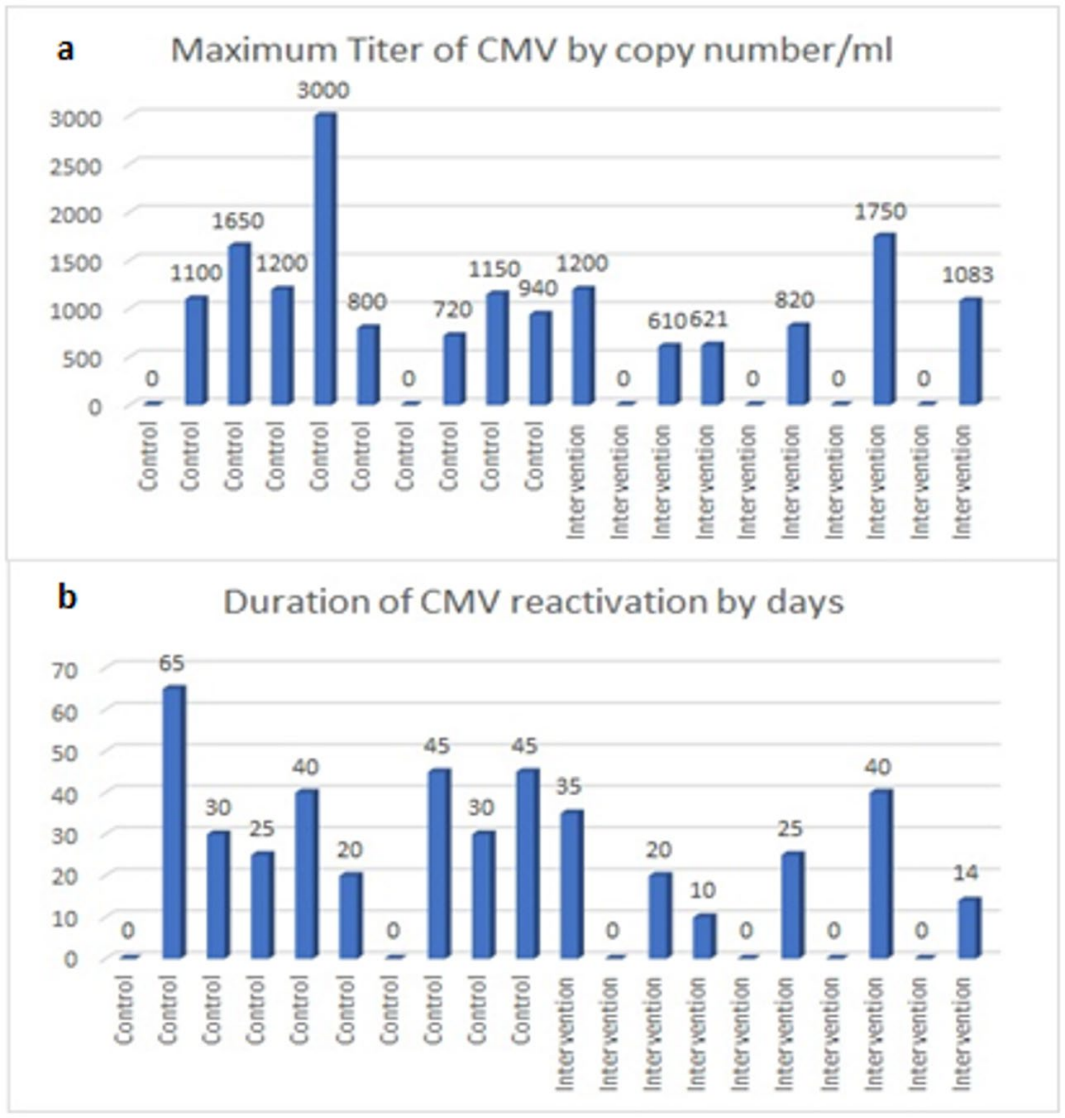
Comparison of maximum CMV titers and duration of CMV reactivation between control and intervention groups. (**a**) Maximum CMV titer (copy number/ml) observed in control and intervention groups. (**b**) Duration of CMV reactivation (in days) for control and intervention groups.

By day 90, CMV reactivation occurred in 52% of the intervention group versus 78% of controls, though this difference was not statistically significant (P = 0.580) (Fig. 2). However, as calculated by repeated-measures analysis, the intervention group demonstrated significantly lower viral loads over time (P = 0.028) (Fig. 3). Peak titers in controls reached 2,500 copies/mL versus 800 copies/mL in the intervention group (Fig. 4a), with reactivation episodes lasting 28 days in controls versus 14 days in the intervention group (Fig. 4b). These durations reflect time to viral clearance under standard preemptive therapy.

### Safety outcomes

CMV-CTL infusions were well-tolerated, with no grade ≥ 3 infusion-related adverse events. Grade II acute GVHD occurred in 30% (3/10) of the intervention group versus 40% (4/10) of controls (Table 2). Mortality rates were comparable between groups (intervention: 20%; control: 30%), with infections and GVHD as leading causes (Table 2).

### Survival and long-term outcomes

With a median follow-up of 14.5 months, overall survival was 80% in the intervention group and 70% in controls. In the intervention arm, one death was due to infection, and the second was associated with severe GVHD. Neither was directly attributed to CMV, while in the control group, two deaths were infection-related, and one was due to relapse (Table 2).

## Discussion

Our findings strengthen the evidence that donor-derived CMV-CTLs are a safe and potential effective strategy for reducing the burden of CMV reactivation after allo-HSCT. While we didn’t see a statistically significant drop in reactivation rates (52% intervention vs. 78% control, P = 0.580, Fig. 2), the intervention group consistently maintained lower viral loads (P = 0.028, Fig. 3)—a finding with real clinical relevance. Peak CMV titers were 3 × lower in treated patients, and reactivation episodes resolved twice as fast (Fig. 4).

These findings are consistent with previous reports demonstrating the antiviral effect of CMV-specific T cells in controlling or preventing CMV viremia^8,12^. The trend toward reduced incidence of CMV reactivation aligns with results from earlier clinical trials, where CMV-CTLs provided rapid restoration of CMV-specific immunity and reduced reliance on antiviral pharmacotherapy^9,11^. While the sample size in our study limits the statistical power, the reduction in CMV burden is clinically meaningful and warrants further investigation in larger, multicenter trials.

## Safety profile of CMV-CTL infusion

In this initial cohort, CMV-CTL infusion exhibited an encouraging safety profile, characterized by the absence of grade 3–5 infusion-related adverse events and overall good tolerability. Acute GvHD of grade II occurred in 30% of patients in the intervention arm and 40% in the control group. This suggests that the infusion of CMV-CTLs did not significantly increase the risk of aGvHD^13^, which is a critical consideration in the context of allo-HSCT. The incidence of aGvHD in both groups is consistent with the expected rates in transplant recipients. Nonetheless, further studies are needed to explore the relationship between CMV-CTL infusion and aGvHD, particularly in larger cohorts and with longer follow-up periods.

## Mechanisms underlying the efficacy of CMV-CTLs

The successful expansion of functional CMV-specific T cells to therapeutic levels further validates the technical feasibility of this strategy. Using overlapping peptide pools derived from immunodominant antigens (pp65 and IE-1), a median 38.9-fold expansion was achieved within 12 days, with a predominant CD3 + population. These findings are consistent with Walter et al. (1995) and support the robustness of current GMP-grade manufacturing protocols^10^. Additionally, the use of CMV-seropositive donors for T cell generation ensures pre-existing immunologic memory, optimizing the antiviral response post-infusion.

## Limitations and future directions

Our study has important limitations. The small cohort (n = 20) and single-center design limit generalizability and power to detect differences in reactivation rates. Additionally, the median follow-up of 14.5 months precludes assessment of long-term outcomes like CMV-specific immunity durability or late-onset GVHD. An important limitation of this interim analysis is that the CMV viral loads in recipients were generally low at the time of intervention, which restricts our ability to fully evaluate the antiviral efficacy of donor-derived CMV-specific CTLs. Future studies in patients with higher viral loads are warranted to fully evaluate the therapeutic potency. Another study limitation is the absence of baseline and serial CMV-specific immune monitoring (e.g., IFN-γ ELISpot, pp65/IE1 multimer, or ICS flow cytometry) due to resource constraints. Future multicenter trials with larger cohorts should validate these findings and explore synergies with antiviral prophylaxis (e.g., letermovir). Incorporating biomarkers (e.g., TCR sequencing) could further refine patient selection for CMV-CTL therapy. Additionally, the use of next-generation sequencing (NGS) to identify novel CMV epitopes could improve the specificity and potency of CMV-CTLs. Advances in gene editing technologies, such as CRISPR/Cas9, may also enable the generation of "off-the-shelf" CMV-CTLs that can be used in a broader patient population^17^.

## Conclusion

Prophylactic CMV-CTL infusion was safe and associated with reduced viral burden in high-risk allo-HSCT recipients. While these preliminary results are promising, the limited sample size and single-center design necessitate validation through larger, multicenter trials to confirm efficacy and long-term safety.

## Supplementary Information

Below is the link to the electronic supplementary material.

Supplementary Material 1

## Data Availability

The datasets generated during and/or analyzed during the current study are available from the corresponding author on reasonable request.
