# Supplementary material for: Prophylactic infusion of donor derived CMV specific T cells for the prevention of CMV reactivation following allogeneic HSCT
Source: Sci Rep. 2025 Dec 4;15:43163. doi: 10.1038/s41598-025-27354-6 (PMC12678814; doi:10.1038/s41598-025-27354-6)
Supplement: Supplementary file 1 — Supplementary Material 1 [file 41598_2025_27354_MOESM1_ESM.docx]

# Study Protocol

**Title**:

Prophylactic Infusion of Donor-Derived CMV-Specific Cytotoxic T Lymphocytes for the Prevention of CMV Reactivation Following Allogeneic HSCT: A Randomized Clinical Trial

## Investigators and Affiliations:

**Principal Investigator**: Dr. Mohammad Ahmadvand

**Institution:** Hematology-Oncology and Stem Cell Transplantation Research Center, Tehran University of Medical Sciences, Iran

## Background and Rationale:

Cytomegalovirus (CMV) reactivation is a significant complication in immunocompromised recipients of allogeneic hematopoietic stem cell transplantation (allo-HSCT). Standard antiviral therapy has limited efficacy and can cause toxicity. Donor-derived CMV-specific cytotoxic T lymphocytes (CMV-CTLs) offer a targeted immunotherapeutic strategy to reconstitute antiviral immunity and potentially reduce CMV reactivation and related morbidity.

:

## Objectives

**Primary Objective:** Assess the safety of prophylactic infusion of donor-derived CMV-CTLs.
**Secondary Objective:** Evaluate the efficacy of CMV-CTLs in reducing CMV reactivation and viral load kinetics after allo-HSCT.

## Study Design

A single-center, randomized, phase I/II clinical trial with two arms:
-Control group: Standard care

- Intervention group: Single intravenous infusion of 10×10⁶ donor-derived CMV-CTLs/m² on day +14 to +21 post-transplant.

## Sample Size

Target: 40 patients (20 per arm). Interim results are reported on the first 20 patients. The sample size was based on detecting a 30% reduction in CMV reactivation with 80% power at α=0.05.

## Inclusion/Exclusion Criteria

**Inclusion Criteria**:
- Adult CMV-seropositive leukemia patients (≥18 years) receiving allo-HSCT from CMV-seropositive donors
- Signed informed consent

**Exclusion Criteria:**
- Unrelated donors
- Concurrent participation in other trials

## Intervention Details

CMV-CTL Preparation:
- Donor PBMCs stimulated with GMP-grade pp65 and IE-1 peptide pools
- Expanded over 12 days with IL-7 and IL-4 in GMP-compliant conditions
- Cryopreserved and infused post-transplant

**Infusion Protocol:**
- 10×10⁶ CMV-CTLs/m² IV as a single dose between days +14 and +21
- Premedication with acetaminophen and diphenhydramine
- No steroids unless for severe adverse events

## GvHD Prophylaxis and Conditioning

Myeloablative conditioning: Busulfan + Cyclophosphamide
GvHD prophylaxis: Cyclosporine + Methotrexate ± ATG/PTCy for haploidentical cases

## Outcome Measures

Primary Endpoint:
- Safety (infusion-related AEs, acute GvHD incidence)

Secondary Endpoints:
- CMV reactivation incidence (quantitative RT-PCR weekly for 3 months)
- Viral load kinetics
- Overall survival

## Statistical Analysis

Mixed-effects model to compare CMV viral loads over time
Cumulative incidence for CMV reactivation
Descriptive statistics and between-group comparisons (STATA v17, GraphPad Prism v9.5.1)

## Ethical Considerations

Approved by Institutional Ethics Committee of Hematology-Oncology and Stem Cell Transplantation Research Center, TUMS (IR.TUMS.HORCSCT.REC.1401.015)
Registered at IRCT (IRCT20140818018842N30) on 18/02/2023
All participants provided informed consent

## Dissemination Plan

Results to be published in peer-reviewed journals and presented at hematology/transplantation conferences.
 Final results will be shared with ethics committee and stakeholders.
